# Sudden arrhythmia in the prone position during spinal surgery: A case report

**DOI:** 10.1097/MD.0000000000030137

**Published:** 2022-08-19

**Authors:** Ji Hyun Kim, Sora Kim, Taeyoung Yu, Woo Seok Yang, Seong Wook Hong

**Affiliations:** a Department of Anesthesiology and Pain Medicine, Kyungpook National University Hospital, Daegu, Republic of Korea.

**Keywords:** arrhythmia, pressure, prone position, spinal stenosis

## Abstract

**Rationale:**

The prone position is the most commonly required position during spinal surgery. Decreasing lumbar lordosis is necessary to facilitate the accessibility of the surgical field. And this can affect the hemodynamic circulation of the patients. The Jackson spine table is one of the most preferred methods, known to have minimal effects on cardiac function.

**Patient concerns:**

We report a case of sudden arrhythmia that developed during the prone position using a Jackson spine table. It occurred 30 minutes after the positional change.

**Diagnoses:**

Arrhythmia showed bizarre P and QRS waves. Ectopic P, bundle branch block, or both was suspected.

**Interventions:**

Because it was difficult to define the exact type or cause of this sudden arrhythmia and considering that other vital signs remained stable, we decided to keep close observation during the operation rather than applying uncertain antiarrhythmic medication.

**Outcomes:**

Arrhythmia spontaneously developed and subsided repeatedly. And it recovered to normal sinus rhythm immediately after the positional change to the supine position. Therefore, increased intrathoracic pressure caused by the prone position was highly suspected to be the cause of this event.

**Lessons:**

Although the Jackson spine table is known to have the least effect on cardiac function, the patient experienced arrhythmia in our case. Hence, to achieve better clinical outcomes, an understanding of physiological alterations and possible complications caused by the prone position is necessary for earlier diagnosis and management.

## 1. Introduction

A prone position is commonly required during spinal surgery. As the curvature of the spine is an important factor in deciding the approachability of the surgical field, decreasing the lordosis of the lumbar spine during prone position is required. However, this change in position can affect both intrathoracic and intraabdominal pressures (IAP), resulting in physiological alterations in the patient. Since Ecker first described^[1]^ 1 type of prone position in 1949, many attempts with different devices have been made to minimize the complications of the prone position. Throughout decades of research, the Jackson spine table has been known as a favorable choice with the least effect on cardiac function and chest wall compliance.^[2]^ Nevertheless, we report a case of sudden arrhythmia that occurred approximately 30 minutes after the prone position using the Jackson spine table. Although arrhythmia spontaneously recurred for 10 to 30 seconds and was restored to normal sinus rhythm, other vital signs were stable during the operation. It returned to the original normal sinus rhythm immediately after we changed the patient to the supine position, and arrhythmia was not observed after. Postoperative electrocardiography (ECG) and echocardiography performed for further workup were unremarkable. Therefore, thoracic compression became the most suspected cause of this event, despite the patient having no chest wall anomalies and even after checking the proper position at the beginning.

## 2. Case report

A 155 cm, 59 kg, 70-year-old woman with spinal stenosis was scheduled to undergo transforaminal lumbar interbody fusion. She had been taking levothyroxine medication for hypothyroidism. Her preoperative thyroid function test showed a euthyroid state with serum T3, free T4, and thyrotropin (TSH) concentrations of 0.88 ng/ml, 1.45 ng/dl, and 0.98 μIU/ml, respectively. Preoperative ECG showed sinus rhythm with heart rate (HR) of 66 bpm. Her routine laboratory results were unremarkable with normal electrolyte levels. Preoperative chest radiography and CT findings were also unremarkable except a tiny nodule in the right upper lung.

General anesthesia was induced and maintained by total intravenous anesthesia using an infusion device (Orchestra Base Primea, Fresenius Kabi, Brezins, France) for effect-site target-controlled infusion of propofol and remifentanil. Standard monitoring, which include ECG, noninvasive blood pressure (BP), oximetry (Radical-7 Pulse Co-Oximeter; Masimo Corporation, Irvine, CA), and patient state index (PSi; Masimo Corporation, Irvine, CA), was applied. The patient’s initial vital signs were stable, with BP, HR, oxygen saturation, and body temperature of 138/86 mm Hg, 76 bpm, 99%, and 36.0°C, respectively. ECG revealed sinus rhythm with intermittent premature atrial complex (PAC). An arterial catheter was cannulated in the right radial artery.

**Figure 1.**
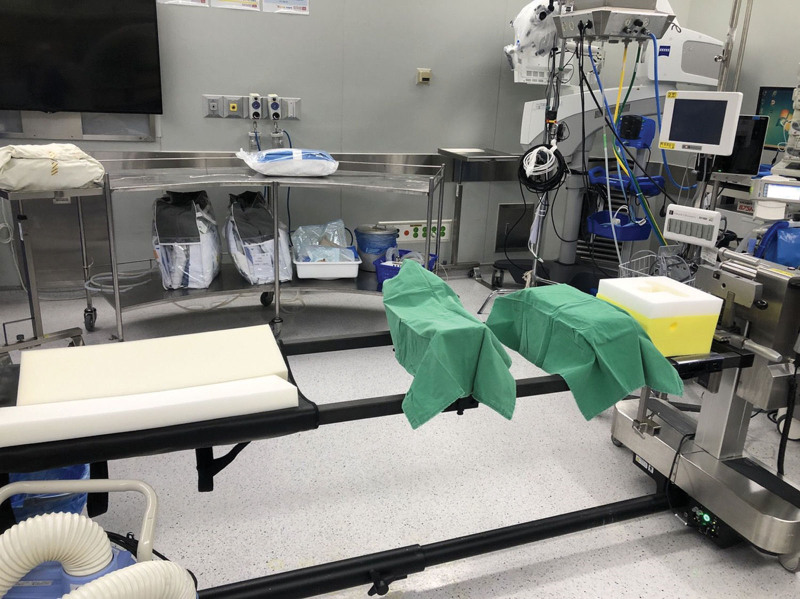
Jackson spine table applied during operation.

**Figure 2.**
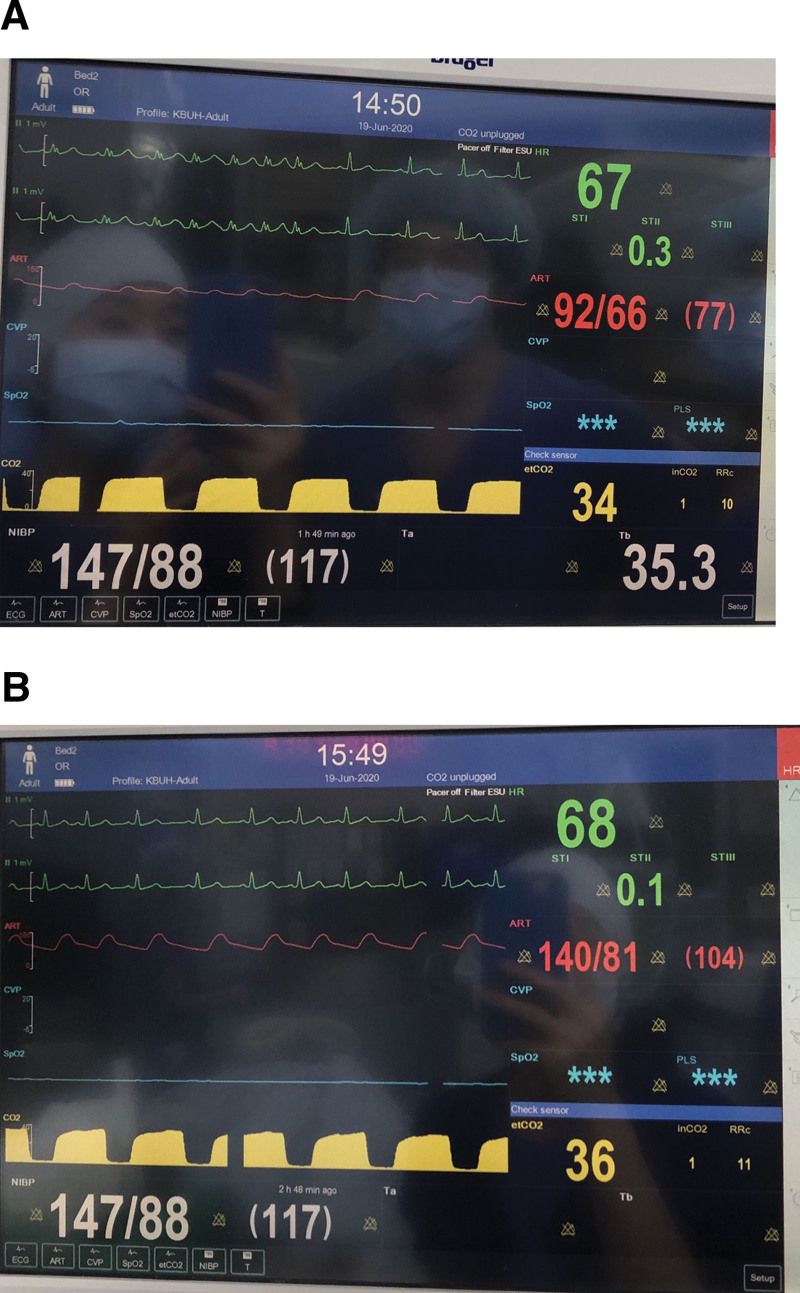
Screen captures of the ECG monitoring device show sudden arrhythmia with bizarre P and QRS wave (A). The ECG restored to original normal sinus rhythm immediately after the positional change from prone to supine (B). Oxygen saturation of the patient is not shown in this monitor because a separate oximetry device (Radical-7 Pulse Co-Oximeter; Masimo Corporation, Irvine, CA, USA) is applied routinely in our clinic.

The Jackson spine table (Mizuho OSI, Union City, CA) was prepared for surgery [Fig. 1]. After the patient’s position was changed from supine to prone, we first confirmed that her body portions were on the right spot, especially the eyes, thorax, and anterior superior iliac spine (ASIS). The ventilator monitor was also checked and demonstrated that there was not much change in peak pressure compared to baseline, only 13 to 15 cmH_2_0. Her vital signs remained stable without any significant changes. However, approximately 30 minutes after positional change, sudden arrhythmia developed [Fig. 2A]. The arrhythmia continued for 10 to 30 seconds and spontaneously restored to normal sinus rhythm for a few minutes. While the event started to recur, no significant changes were observed in other vital signs. BP and HR were maintained at approximately 95/60 mm Hg and 65 to 75 bpm, respectively. Arterial blood gas analysis showed unremarkable electrolyte values, with ionized sodium, potassium, and calcium concentrations of 141, 3.7, and 1.02 mmol/L, respectively. The arrhythmia initially showed a p wave with bizarre-widened QRS wave, which appeared as an rsR shape at first. Subsequently, the p wave became slightly irregular, and eventually, it was hidden in the T wave. Therefore, we could assume that it was an ectopic p wave, bundle branch block or both mixed. As it was uncertain to diagnose the clear pattern and considering that the vital signs remained stable, we decided to hold any reckless challenge of antiarrhythmic medication. Instead, we maintained close observations for the time and the phenomenon continued approximately 2 hours.

However, at the end of the operation, the ECG pattern was restored to original normal sinus rhythm, immediately after the positional change to the supine position [Fig. 2B]. No additional arrhythmia pattern was observed during emergence, and it remained unremarkable in the recovery room. The patient showed no postoperative cardiac symptoms. Postoperative ECG at the ward also showed normal sinus rhythm. On postoperative day 6, transthoracic echocardiography was performed and showed an ejection fraction of 56% (55–70%), ratio of mitral peak velocity of early filling to early diastolic mitral annular velocity (E/E’ ratio) of 7.96 (>8, <15) and mild tricuspid regurgitation with right ventricular systolic pressure of 39 mm Hg (15–45 mm Hg for those 50–75 years of age). The patient’s preoperative chest CT was relooked to find whether if we missed any chest anomaly which can be affected by prone position. We measured Haller index to assess anteroposterior diameter of the chest cavity and the result showed 2.01 which can be considered as nearly normal (defined as < 2). The patient was discharged without any surgical or cardiac sequelae. However, 2 years later, on her regular postoperative checkup, her follow-up ECG which was performed due to her previous cardiac event, showed left bundle branch block. Her echocardiography result was similar to those before and she had no cardiac symptom. The cardiologist was consulted and suggested the patient for further evaluation, but the patient refused to do so.

## 3. Discussion

As Yokohama et al reported,^[3]^ the flat prone position itself does not result in significant changes in cardiovascular values. However, from the surgeon’s perspective, it is inevitable to decrease lordosis of the lumbar spine to have better access. Therefore, hemodynamic circulation is interfered when we attempted to change the curvature of the spine with devices. As we develop lumbar kyphosis, increased IAP becomes one of the most important factors for increased surgical bleeding because of the valveless communication between inferior vena cava (IVC) and vertebral veins. Kneeling position in the 1949 study by Ecker^[1]^ was one of the first described extreme measures to lower IAP. However, as venous pooling at the lower limbs can result in serious decrease in preload, this is not considered as the best option. Since then, several attempts have been studied over the past decades.

For anesthesiologists, IAP alone is not the only physiologic alteration to consider in the prone position. A decrease in cardiac index (CI) is an almost universal finding in the prone position,^[4,5]^ although a few studies have shown no change in CI.^[6,7]^ A decreased CI reflects a decrease in stroke volume (SV), which can be explained in several ways. First, although it could vary depending on which support system we choose to apply, compressed IVC or venous pooling due to extremities positioned below the heart can result in a reduction of venous return (VR) and SV. Meanwhile, the thorax is held on by bolsters or pads in various ways. Using echocardiography, Toyota et al^[6]^ demonstrated a significant reduction in end-systolic and end-diastolic left ventricular area and volume, which defines decreased left ventricular compliance related to increased intrathoracic pressure.

After comparing 5 different types of prone position with echocardiography, Dharmavaram et al^[2]^ concluded that the Jackson spine table or longitudinal bolsters table had the least effect on hemodynamic circulation. And these are the most preferred spine tables for lumbar spine surgery these days. Both the Jackson and longitudinal bolsters tables have the advantage of adequate venous return as they maintain the lower extremities at the heart level. The difference between them comes from creating lumbar kyphosis. In this process, “longitudinal bolsters” table literally use bolsters longitudinally at the lateral sides of the whole trunk from chest to pelvic area. Meanwhile, the Jackson spine table has a separate transverse pad for supporting the thorax from the ASIS supporting pad; consequently, the abdomen area is allowed to be hung freely, lowering IAP even more.

However, the Jackson spine table has some weakness in using a transverse pad at the thorax when the patient has chest wall anomalies.^[8,9]^ Alexianu et al^[8]^ reported the case of a patient with posterior mediastinal neurofibromatosis, pectus excavatum, and scoliosis. The patient showed hemodynamic collapse soon after prone positioning as his right ventricle was compressed by the sternum. They could resolve pressure on the sternum by changing the type of bolster from transverse to longitudinal. Neira et al^[9]^ also demonstrated with echocardiography that even right ventricular outflow tract obstruction can occur with a transverse bolster if the patient has chest deformity. The narrowed anteroposterior diameter of the chest is vulnerable to compression in the prone position, and a transverse bolster seems to aggravate this pressure. In such cases, longitudinal bolsters could be useful substitutes for releasing this pressure. In our case, even though the patient had nearly normal value of anteroposterior thoracic diameter and no other anomalies, thoracic compression by transverse pad is suspected to be the main cause for arrhythmia. Therefore we assume that switching to longitudinal bolsters would have been helpful to resolve the problem we experienced.

Looking back, it was challenging for us to clearly define a certain type of arrhythmia with restrictive leads in the operating room, which delayed detecting the main cause of the event. Even though the patient showed intermittent PAC at the beginning of anesthesia, it seemed less likely to cause sudden further arrhythmia. Because PAC is a benign sign per se, which can occur even with anxious state and it actually disappeared after induction phase in our case. Arrhythmia appeared to be ectopic beat and bundle branch block with bizarre p and QRS waves. Considering that HR did not increased and maintained stable around 65–75 bpm, it was difficult to see that PAC alone is related with other conduction issue such as bypass or reentry tract. Even though it was difficult to determine the exact rhythm, intraoperative transesophageal echocardiography might have been helpful. However, it is not routinely performed during the prone position because of its risk of recurrent laryngeal nerve injury. A Vigileo monitor with a FloTrac sensor (Edwards Lifesciences LLC; Irvine, CA) also could have been a very useful tool to evaluate further hemodynamic changes. If we checked the reduction of CI or SV when arrhythmia appeared, we could correlate those values, which might help us to suspect mediastinal compression more promptly.

The reason why her chest was stressed enough to develop arrhythmia still remains in question. We assume that there was a slight change in position after we first checked. Alternatively, as development of left bundle branch block was incidentally found at the follow-up check, we cannot exclude that this was the result of this event; however, on the contrary, she might have unknown nodal or bundle branch block issues before surgery. Pump et al^[7]^ suggested the theoretical possibility that decreased arterial filling caused by thoracic compression can lead to enhanced sympathetic nervous activity which can be hazardous for patients with cardiac disease. In addition, Shimizu et al^[10]^ analyzed cardiac function changes in prone position using quantitative semiconductor gated single-photon emission computed tomography. According to their research, the prone position induces significant changes in both systolic and diastolic functions as well as dyssynchrony of the heart. It is more severe in patients with a poor baseline cardiac function.

In our case, even without chest anomaly or previous cardiac history, and using one of the least effects on the cardiovascular table, the patient experienced arrhythmia during the prone position. We assume that if we had better understanding of the hemodynamic alterations of the Jackson spine table, we could have tried to recheck or change the types of bolsters earlier. Although the popularity of the prone position is increased, physiologic differences depending on the types of spine table remain unfamiliar. Considering there are various prone spine tables routinely used in each centers, it is important for anesthesiologists to reevaluate and comprehend the benefits and risks of each table for better clinical outcome.

## Author contributions

Conceptualization: Ji Hyun Kim.

Data curation: Seong Wook Hong.

Investigation: Sora Kim, Taeyoung Yu.

Resources: Woo Seok Yang.

Writing - original draft: Ji Hyun Kim.

Writing - review & editing: Ji Hyun Kim.
